# Prevention of Peripheral Distal Polyneuropathy in Patients with Diabetes: A Systematic Review

**DOI:** 10.3390/jcm11061723

**Published:** 2022-03-21

**Authors:** Lidia Carvajal-Moreno, Manuel Coheña-Jiménez, Irene García-Ventura, Manuel Pabón-Carrasco, Ana Juana Pérez-Belloso

**Affiliations:** 1 Department of Podiatry, University of Seville, 41009 Seville, Spain; lidiacarvajalmoreno@gmail.com (L.C.-M.); irenepie1999@gmail.com (I.G.-V.); aperez30@us.es (A.J.P.-B.); 2 Spanish Red Cross Nursing School, University of Seville, Avda. de la Cruz Roja, nº 1 Dpdo., 41009 Seville, Spain; mpabon@cruzroja.es

**Keywords:** diabetes mellitus, diabetic complications, diabetic neuropathy, prevention and control, evidence, systematic review

## Abstract

Background: Diabetic peripheral neuropathy (DPN) is the most frequent chronic complication and is that which generates the highest disability and mortality in diabetes mellitus (DM). As it is currently the only microvascular complication of DM without a specific treatment, prevention is essential. The aim of this study was to determine the most effective preventive strategy to avoid or delay the appearance and/or development of DPN in patients with DM. Methods: A systematic search was carried out in the main health science databases (PubMed, Scopus, CINAHL, PEDro and The Cochrane Library) from 1 January 2010 to 31 August 2020. The study selection was conducted by two independent reviewers and data extraction was performed by the author. The eligibility criteria included randomized clinical trials (RCTs) and cohort studies from RCTs. Results: Eleven studies were selected that included 23,595 participants with DM. The interventions evaluated were intensive or standard glycemic control, the use of drugs to achieve glycemic control, and the promotion of a healthy lifestyle and exercise. Intensive glucose control achieved a significant reduction in the development of DPN in TIDM patients, and lifestyle modifications and exercise achieved it moderately in TIIDM patients. Conclusions: The main preventive strategy for DPN is intensive glycemic control with a target HbA1c < 6% in patients with TIDM and standard control of 7.0–7.9 in patients with TIIDM, incorporating lifestyle modifications.

## 1. Introduction

Diabetic neuropathy (DN) is the most frequent chronic complication in diabetes mellitus (DM) [1,2,3,4], and is considered the most important predictor of mortality in patients with type II diabetes (TIIDM), being currently the only microvascular complication of DM without specific treatment [5]. Diabetic peripheral neuropathy (DPN) is the most common cause of diabetic foot complications, with chronic sensorimotor symptoms and signs [1]. There are several forms of DPN. The most common type is distal symmetric polyneuropathy, which causes neuropathic pain symptoms. Atypical forms of DPN include mononeuritis multiplex, radiculopathies, and treatment-induced neuropathies. Other diabetic neuropathies include autonomic neuropathies that affect the cardiovascular, gastrointestinal, and urogenital systems [5,6]. Due to the lack of treatments targeting the underlying nerve damage, prevention is the key component in this complication of DM, and for this reason it is essential to emphasize special attention paid to the feet, as these patients are at risk of injury due to a lack of sensation [6,7,8].

In this sense, diabetic foot is considered one of the conditions that generates more disability, economic costs in health systems and mortality [9]. It may be considered as a supercomplication of several complications. Thus, patients with DM have a high rate of lower limb amputation, which increases when DN is present, and consequently the risk of foot ulceration is three times higher in patients with DN [10,11,12,13]. This complication in the lower extremities can be life-threatening in patients with foot ulceration, and can lead to subsequent infection. In this sense, since most amputations are preceded by foot ulceration, infection must be avoided. More extensive research is necessary for determining more precisely the need for amputation. It is important to avoid non-painful foot injuries by wearing well-fitting footwear and by performing regular inspections [4,6]. Health education is essential. DPN is the most common form of DN; its presentation is slow and progressive, usually distal and symmetrical. There is a progressive loss of sensitivity as well as motor weakness of the affected muscles, and dysfunction of the peripheral nerves of the autonomic nervous system, acting mainly on the lower limbs. Patients often report a sensation of “numb” feet, have altered distal vibratory sensation as well as altered joint position and sensations of tactile pressure and abnormal reflexes [12]. Normally, none of these alterations are painful, although it is reported that up to 25% of these patients may experience symptoms of neuropathic pain. It is described as numbness, paresthesias, hyperesthesias, allodynia, loss of sensation, muscle weakness, or loss of temperature sensation, risk of the complications of diabetic ulceration and non-traumatic amputation [3]. Amputation decisions are determined by patient comorbidities, performance, imaging studies, and clinical examination results [7,8]. In this sense, more extensive research is necessary to determine more precisely the need for amputation. 

The most important risk factor for the development of this complication, apart from the duration of the disease, is hyperglycemia [14]. Intensive control is associated with a reduction in the prevalence of DN and painful symptomatology, especially in patients with type I DM (TIDM). In the case of patients with type II DM (TIIDM), good glycemic control is recommended in addition to the control of cardiovascular risk factors and lifestyle modifications [15,16,17,18,19]. 

Some studies reported that screening for symptoms and signs is very important, as it allows for early diagnosis in the early stages of DN [20]. It is estimated that about half of patients with DM are undiagnosed [21], and it is also established that the group of patients with glucose intolerance and prediabetes may also develop neuropathies, mainly DPN, as this is the most common form of presentation [11]. In addition, it is stated that up to 50% of patients with DPN may be asymptomatic [8]. DPN affects at least 20% of patients with TIDM, 20 years after disease onset, and 10–15% of newly diagnosed patients with TIIDM, increasing to 50% 10 years after diagnosis [20]. Of these patients, 10–15% may develop painful DPN, and symptomatic treatment may be necessary. Painful symptoms, as well as other types of complications derived from DPN, can have a significant impact on the quality of life of these patients. In addition, patients with DM with pain have three times the expenditure on medication, so in this sense, prevention is essential [14], considering that the expenditure on medication is expensive to health systems [1,9].

On the other hand, early diagnosis, prevention and treatment of symptoms help to reduce sequelae, costs and improve the quality of life of patients with DN. Despite a large body of evidence, current medication prescribing patterns are inconsistent. Previous studies reported first-line drugs for the treatment of neuropathic pain in painful DPN, including the α-2-delta subunit voltage-gated calcium channel blockers gabapentin and pregabalin, the selective serotonin and norepinephrine reuptake inhibitors (SNRIs) duloxetine, and the tricyclic antidepressant (TCA) amitriptyline. The most studied drug, and with the most beneficial results, is pregabalin [15,22]. Thus, the American Diabetes Association (ADA) recommends starting symptomatic treatment of neuropathic pain in DM with pregabalin or duloxetine, although gabapentin can also be used, but the patient’s socioeconomic status, comorbidities, and possible drug interactions must be taken into account [7]. Opioid and atypical opioid analgesics are associated with a high risk of addiction and safety concerns and numerous serious adverse effects such as abuse or mortality. To date, prevention of DN has focused primarily on glycemic control [19,22]. Although studies have been published that point out other types of preventive strategies to avoid the onset, development and evolution of this complication of DM, these lack great scientific evidence due to the poor quality of the studies, and on numerous occasions provide confusing results [7]. In this sense, this research attempts to shed light on the existing preventive alternatives for DN, not only highlighting the role of glycemic control as a preventive factor, but also revealing other options.

In view of these considerations, the aim of the present review was to determine which was the most effective preventive strategy to avoid or delay the appearance and/or development of DPN in patients with DM.

## 2. Materials and Methods

### 2.1. Protocol and Registration

This systematic review was carried out according to the general guidelines and recommendations made by the Preferred Reporting Items for Systematic Reviews and Meta-Analyses (PRISMA) and was registered in the PROSPERO database (CRD:42020206120).

### 2.2. Eligibility Criteria

The study population consisted of patients with DPN. Documents published up to 30 September 2021 were included. We excluded documents that did not meet the eligibility criteria and those dealing with the diagnosis of DPN, studies on gestational diabetes and on the treatment of painful DPN, and investigations related to any neuropathy other than DPN. Documents that were not published in English, Spanish, French or Portuguese were excluded. Cohort studies and RCTs carried out from 1 January 2010 to 31 August 2020, following the PICO strategy.

1. Participants: Patients with DM, aged ≥ 18 years.
2. Interventions: Any strategy that entailed prevention or delay of DPN onset.
3. Comparisons: Placebo substances, any other alternative or natural progression of the disease in the control group.
4. Outcomes or results: The effectiveness of the intervention in terms of the prevention of DPN at the end of the studies in patients who did not present this condition at the beginning, or the improvement of this condition if they presented it at the beginning of the study, should be evaluated. Other outcomes may include quality of life measurements, adverse events, related costs, changes in neuropathic pain symptoms, presence of foot ulcerations and/or amputations, and events that prevented continuation of clinical trials.

### 2.3. Sources and Search

The databases used were Scopus, Cochrane, PubMed, PEDro, EMBASE, SciELO and CINAHL. PubMed was used as a free access tool for the search in Medline and Premedline. The search and the free search were done via Mesh terms. The following search terms were used, together with the operators “OR” and “AND”. According to each database, the following search strategy was used. The key words used for the search were “diabetic neuropathies”, “prevention”, “control”, “wound”, “randomized controlled trial”, “diabetic nephropathy”, “case control studies”, “quality of life”, “cerebrovascular accident”, “cardiovascular disease”, “diabetic nephropathies”, “peripheral occlusive artery disease”, “autonomic neuropathy”, “coronary artery disease”, “depression”, “neuropathic pain”, “healthcare cost”, and “diabetic retinopathy”. The search strategy used can be consulted in Appendix A.

### 2.4. Study Selection

Two blinded reviewers (XXX) (XXX) participated in each stage of the study selection. First, they screened by titles and abstracts of the references identified through the search strategy. The authors assessed whether the studies collected through the literature search met the eligibility criteria, excluding those that were irrelevant and/or whose level of methodological quality was questionable. Full reports of all potentially relevant documents were then assessed for eligibility based on the eligibility criteria of this review. Disagreements were resolved by discussion between the two evaluators, or if consensus was not possible, further opinion was sought (XXX) (XXXX).

### 2.5. Data Extraction and Synthesis of Results

For the data extraction process, review authors used a standardized template containing information related to the eligibility criteria of the publications and the exclusion reasons for the selection of articles, and full title, country, and year of publication. After carrying out the first evaluation of the reports, the results obtained were discussed between the investigators, as well as the inclusion or exclusion of incompatible papers and, if necessary, the intervention of a third independent investigator. Finally, a form was designed for the extraction of data from the articles ultimately selected. This task was carried out by a single researcher. The data extracted were synthesized in an evidence table (including study design and setting, population characteristics, risk of bias assessment).

### 2.6. Risk of Bias Assessment

The assessment of the risk of bias in the studies was carried out using the Review Manager tool (RevMan) of the Cochrane Collaboration, version 5.3.77. This software evaluates the risk of bias of individual studies as well as among the studies included in the review by generating graphs, tables and percentages from the following domains.

The risks of bias criteria are classified as: “low risk”, “high risk” or “unclear risk”, assessing the risks of selection, conduct, detection, attrition, reporting and other possible biases. This task was carried out by the review author and is currently the main tool used for the assessment of risk of bias in studies and for the evaluation of methodological quality [23]. Thus, studies without a high risk of bias in any category were considered to be of high quality (1++), and those with a high risk or two unclear risks were considered to be of medium quality (1+). The rest were considered low quality (1−).

In addition, the STROBE [24] and CASPe [25] checklists were used to assess the quality of cohort studies and RCTs, respectively. These two methodological quality assessment scales are expressed as a numerical score based on the number of items completed. A statistical assessment was performed by two independent assessors using the IBM SPSS Statistics 22 80 software. The data were analyzed using the intraclass correlation coefficient (ICC), the purpose of which is to assess the agreement between two or more continuous measurements carried out repeatedly in a sample. The ICC takes values between 0 and 1. A significance level of less than 0.04 would indicate poor reliability, and values above 0.75 would indicate excellent reproducibility; intermediate values are considered adequate.

## 3. Results

**Figure 1:**
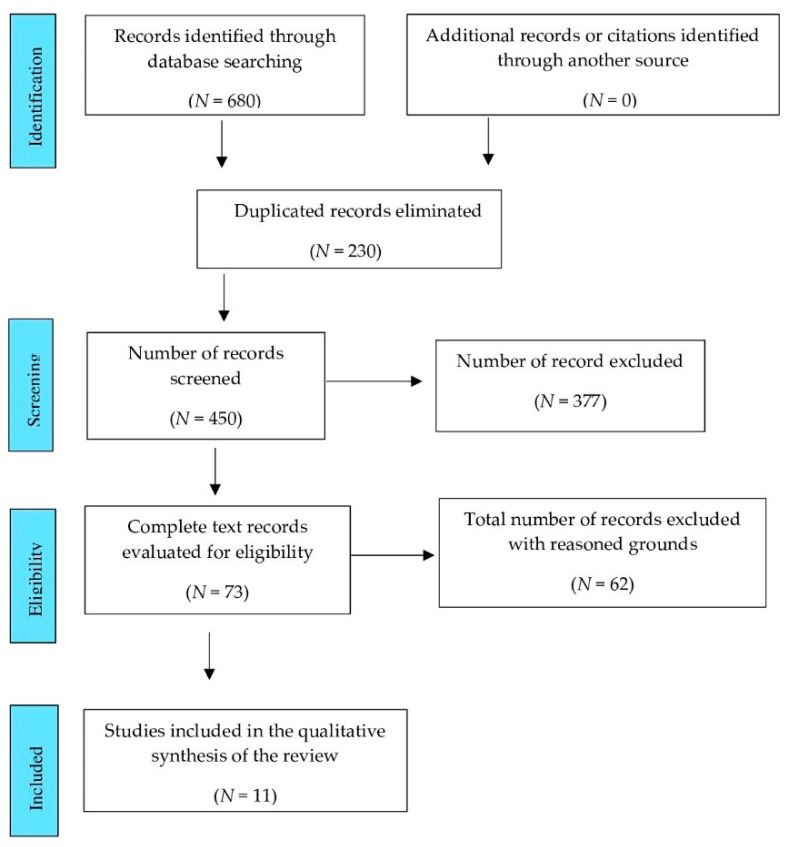
PRISMA flow diagram adapted with permission from the PRISMA group, 2020.

The flow diagram summarizes the study selection processes, and each stage for the studies included in this review (see for details the PRISMA flow diagram in Figure 1) [26]. In total, 11 documents were included in our systematic review. Table 1 shows the studies excluded and the reasons after the application of the quality appraisal filter.

### 3.1. Risk of Biases among the Studies Included

**Figure 2:**
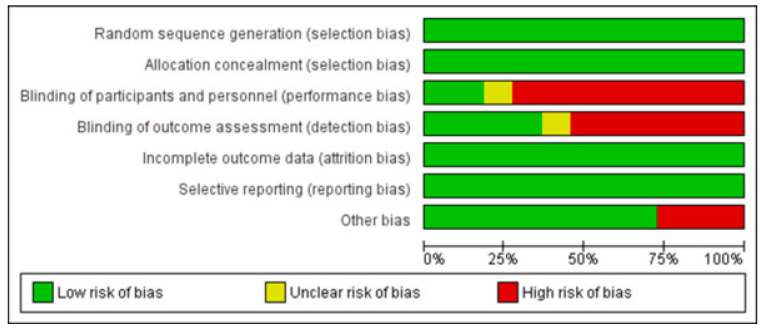
Risk of biases of included studies, overall analysis.

**Figure 3:**
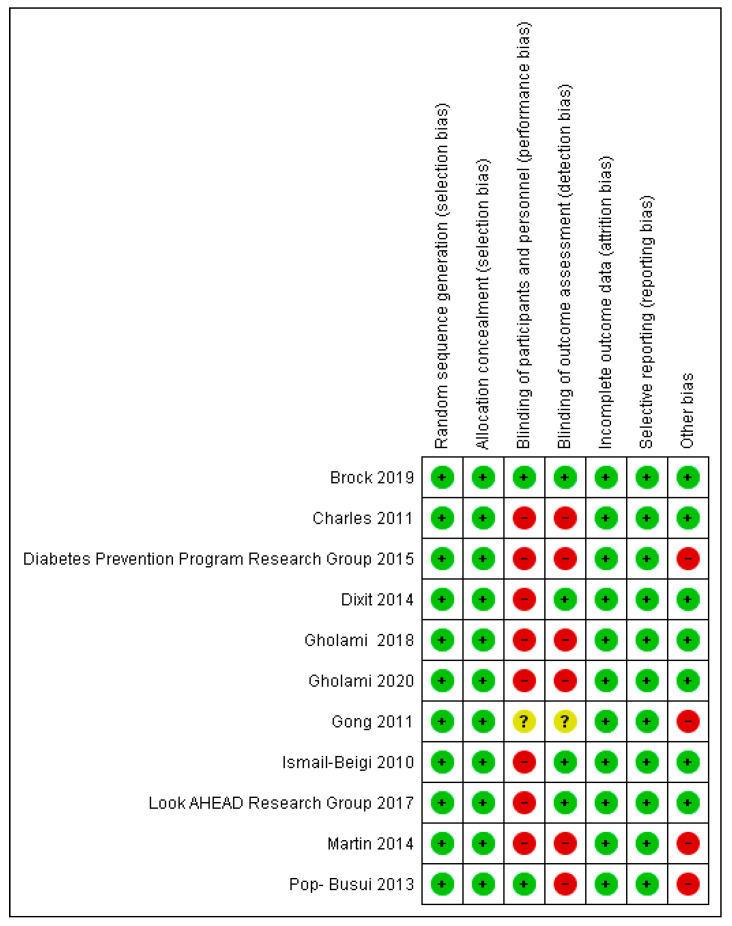
Risk of biases of the included studies [15,53,54,55,56,57,58,59,60,61,62], individual analysis. Green: low risk, yellow: unclear risk, red: high risk.

Figure 2 and Figure 3 show the risk of biases of the study included in this systematic review.

Allocation concealment and random sequence generation was evident in 100% of the studies. Blinding of participants and staff was present in less than 25%, and blinding of assessors was present in less than 50% of the included articles. Due to the nature of some included studies, such as cohort studies, 25% of the included studies were considered to be at high risk of other biases.

The levels of evidence evaluated according to the quality of the selected articles received a score of 1++ in 9.2% (*n* = 1) [53] qualifying it as high quality, 27.3% of the studies received a score of 1+ or medium quality (*n* = 3) [54,55,56], and the rest of the articles were scored as low quality, 1−, representing 63.5% (*n* = 7) [15,57,58,59,60,61,62].

### 3.2. Statistical Analysis of the Quality of the Included Studies

Detailed assessment ICC is summarized in Table 2. Table 3 summarizes the scores of the quality scales of the studies included in this review. The limitations of the review are summarized in Table 4.

### 3.3. Limitations of Included Studies

Table 4 shows some of the studies with their limitations. Some of the reasons for its limitation were the sample size, number of dropouts or that not all patients were evaluated with all the measures, among other reasons.

**Table 4:** Limitations of the review.

Authors	Limitations
Ismail-Beigi et al., 2010	Early termination of the RCT due to increased mortality among participants.
Charles et al., 2011	Not all patients were evaluated with all measurements. Patients in the CASE IV subgroup were younger than the rest, so microvascular complications may have been lower in this group.
Gong et al., 2011	No results were obtained for 25% of the participants who died. Low incidence of nephropathy and neuropathy due to short duration of diabetes in participants.
Pop-Busui et al., 2013	Study not designed to detect an effect of the groups on DPN. A lower incidence of neuropathy was found in the IS group; however, the authors were unable to identify whether the benefit was specific to biguanides or thiazolidinediones. Small fiber neuropathy was not evaluated, as only the Michigan Neuropathy Screening Instrument (MNSI), which evaluates large fibers, was used. Subjectivity of the MNSI.
Dixit et al., 2014	The effect of aerobic exercise to halt or interrupt the natural course of DPN was not studied. The study had a large number of dropouts.
Martin et al., 2014	Intentional exclusion at the start of Diabetes Control and Complications Trial (DDCT) of participants with severe neuropathy. Patients in the conventional insulin therapy (CON) group were switched to intensive insulin therapy (INT) group because of the benefits of intensive glycemic control in patients with TIDM.
Diabetes Prevention Program Research Group et al., 2015	The combination of three different microvascular outcomes in the aggregate microvascular outcome.
Look AHEAD Research Group et al., 2017	Relationship of biguanide use with vitamin B12 depletion and the development of DPN. Levels of this vitamin were not recorded. Diagnosis of DPN by questionnaire, MNSI physical examination and Semmes-Weinstein (SW) monofilament.
Gholami et al., 2018	Small sample size, large number of dropouts, and only male participation.
Brock et al., 2019	Severe irreversible neuropathy, more male representation.
Gholami et al., 2020	Small sample size.

### 3.4. Synthesis of Results

#### 3.4.1. Studies Included

Of the 11 included studies, seven were parallel-group RCTs [59,61], of which one was placebo-controlled [53]. The remaining four studies were cohort studies from RCTs, [58,60], of which one was placebo-controlled. The total follow-up period of the studies ranged from 8 weeks to 20 years. Table 5 summarizes the characteristics of the included studies.

#### 3.4.2. Participants

The total number of participants in all studies was 23,595, with ages ranging from 33.6 ± 7 to 66.7 ± 9.2 years, including 1834 patients with TIDM and 21,761 patients with TIIDM [54,55,56,57,58,59,61,62]. All studies divided participants into two groups, except the 2015 Diabetes Prevention Program Research Group et al. [57] study, which randomized participants into two intervention groups and one control group.

#### 3.4.3. Interventions and Comparisons

Interventions included drugs such as liraglutide [53] for the reduction in the neuroinflammatory component that appears in DPN in patients with TIDM, intensive glucose control with a glycosylated hemoglobin (HbA1c) < 6% in the case of patients with TIDM [15], or in patients with TIIDM [55,62]. Another strategy employed was the comparison of insulin-sensitizing treatments and insulin-providing treatments for standard glycemic control in patients with TIIDM [60]. Moderate aerobic exercise was evaluated in two of the included articles [54,61], as well as cycling [59]. The most employed intervention among the included studies was the promotion of a healthy lifestyle through education, medication for the control of diabetes and cardiovascular risk factors in addition to diet in patients with TIIDM [56,57,58]. Comparisons were made with placebo [53,57], standard recommendations for diabetes care [60,62], maintaining usual physical activity level [59,61], diabetes education focused on exercise and diet control [56].

#### 3.4.4. Analysis of Results

The presence of DPN was mainty evaluated. Other variables were taken into account, such as ankle arm index (AAI) [58,62], albuminuria and creatinine (nephropathy), fundus examinations [58] (retinopathy), glucose levels [59], oral glucose tolerance test [58], HbA1c [62], lower limb inspection [58], weight, body mass index (BMI), fat percentage [61], diagnosis of DM [57] or changes in intima media thickness and basal diameter of the superficial femoral artery [59]. In the case of neuropathy identification, the measurements used were nerve conduction velocity (NCV) studies [15,53,54,59,61], tests for vibration detection threshold assessment with a 128 Hz tuning fork, and light touch with the SW monofilament [58,62], and questionnaires such as the Michigan Diabetic Neuropatic Score (MDNS) [54,59] or the MNSI [55,56,60]. For all the results obtained in the studies, the significance level was *p* < 0.05.

#### 3.4.5. Summary of Results

The drug liraglutide reduced the neuroinflammatory component interleukin-6 in adults with TIDM, but did not improve established DPN [53]. Intensive glycemic control significantly reduced the development of neuropathy in patients with TIDM, but this effect was not observed in patients with TIIDM [55]. Intensive lifestyle intervention in patients with TIIDM had negative effects in two of the studies [57,58], and positive effects in one [56]. Moderate-intensity aerobic exercise had a positive outcome for the improvement of established DPN and prevention in two of the included studies [54,61], as did cycling in patients with TIIDM [59]. Glycemic control therapy with insulin sensitizers significantly reduced the incidence of DPN compared with insulin-providing therapy, with more benefits for men [60]. The effect of glycemic control therapy with insulin sensitizers in patients with TIIDM was not observed [61,62].

## 4. Discussion

The aim of this systematic review was to determine which is the most effective preventive strategy to avoid or delay the appearance and/or development of DPN in patients with DM. Most studies seem to indicate that glycemic control is currently the most effective preventive strategy. Our literature search identified 11 studies examining patients with the variables related to diabetic neurophaties [15,53,54,55,56,57,58,59,60,61]. These aims were achieved in the review.

### 4.1. Intensive Glycemic Control

DPN has a multifactorial origin, in which different metabolic, inflammatory, autoimmune and vascular processes take place, leading to nerve degeneration [62]., Therefore, the prevention of these alterations is fundamental, with the control of maintained hyperglycemia being the main one [63]. In this sense, large studies have been carried out in which the effect of intensive glucose control with a target HbA1c of less than 6% in patients with TIDM were evaluated [64].

The Epidemiology of Diabetes Interventions and Complications (EDIC) study was performed to record the long-term effects of therapy on the development and progression of myocardiovascular complications and cardiovascular disease. Data published in 2010 by Albers et al. [65] from the EDIC follow-up demonstrated that intensive glucose control significantly delayed the development and progression of DPN. The prevalence of neuropathy increased from 9 to 25% in the INT group and from 17 to 35% in the conventional CON insulin therapy group (*p* = 0.001) and the incidence also remained lower in the INT group (22%) relative to the CON group (28%); (*p* = 0.0125). The effect was maintained in the article included in our 2014 systematic review of Martin et al. [15] in which the prevalence and incidence of DPN and Cardiovascular autonomic neuropathy (CAN) remained significantly lower in the Diabetes Control and Complications Trial (DCCT) intensive therapy group compared to the DCCT conventional therapy group up to year 13/14 of EDIC. This is in addition to being maintained in other smaller European cohorts, such as the Oslo study [66], and the one published by Ziegler et al. [67] in 2015, as well as in the EURO-DIAB study [68]. In contrast, the results presented by Holman et al. [69] in 2008 of the 10-year follow-up of participants in the United Kingdom Prospective Diabetes Study (UKPDS) in the sulfonylureas-insulin group, relative risk reductions persisted for microvascular disease (*p* = 0.04), but this effect was not seen in the metformin group of patients with TIIDM. Along the same lines, the Steno-2 study, according to data published by Gaede et al. [70] in 2003, did not have a significant effect on the progression of DPN after a follow-up of 13.3 years in patients with microalbuminuria, although it did reduce the development of CAN by 57% (Relative risk; RR 0.37; Confidence interval, 95% CI 0.18–0.79). With similar results, the 2008 ADVANCE study [71], which included 11,140 patients with TIIDM, also with two groups, one intensive therapy and one conventional glycemic therapy, demonstrated a decrease in the incidence of combined major macrovascular and microvascular events (*p* = 0.01), as well as in major microvascular events (*p* = 0.01), mainly due to the reduction in the incidence of nephropathy (*p* = 0.006), but did not demonstrate a significant difference in the groups in terms of relative risk reduction for the occurrence of DPN.

The Action to Control Cardiovascular Risk in Diabetes (ACCORD) trial [72] was an RCT published in 2008 that studied the relationship between diabetes and cardiovascular disease, concluding that, compared with standard therapy, the use of intensive therapy to achieve target HbA1c levels for 3.5 years increased mortality and did not significantly reduce major cardiovascular events, which is why standard glycemic therapy rather than intensive therapy is advised in patients with TIIDM. In the ACCORD results for the development of microvascular complications presented in the 2010 study by Ismail-Beigi et al. [55], positive results were obtained for intensive therapy in terms of DPN prevention, but due to the increase in mortality and the number of cardiovascular events recorded, this study advises against intensive glycemic control in patients with TIIDM. Similarly, in the 2009 Veterans Affairs Diabetes Trial (VADT) RCT [73], no difference was found between the intensive or standard glucose control groups for microvascular complications of DPN after a median follow-up of 5.6 years.

In addition, the multicenter Anglo-Danish-Dutch Study of Intensive Treatment in People with Screen-Detected Diabetes in Primary Care (ADDITION-Denmark) study by Charles et al. [62] published in 2011 did not find that screening followed by intensive glycemic control intervention led to a statistically significant difference in the prevalence of DPN and peripheral arterial disease (PAD) 6 years after diagnosis. However, positive results have been obtained for intensive control in patients with TIIDM in a Japanese RCT with a small sample size, significant improvement in NCV (*p* < 0.05) and vibration thresholds (*p* < 0.05) at 6 years from the baseline [74]. In this line, in 2013, Hur et al. [43], performed a cohort study where they identified that HbA1c levels predict nerve degeneration and regeneration of myelinated fibers in patients with TIIDM and DPN. Therefore, maintaining optimal blood glucose control is likely to be essential to prevent nerve injury. Abraham et al. [51] 2017, Ishibashi et al. [45] 2019 and Cho et al. [44] 2014 further added the importance of dyslipidemia control, as high cholesterol and triglycerides seem to be found to be related to the future development of DPN in patients with TIIDM.

In 2012, a Cochrane review and meta-analysis by Callaghan et al. [75] was published that aimed to examine the evidence for intensive glucose control in the prevention of DPN in patients with TIDM and TIIDM. Revealing a significant decrease in the relative risk of developing clinical neuropathy in those who had intensive glucose control, RR of −1.84% (95% CI −1.11 to −2.56). For patients with TIIDM, the relative risk of developing neuropathy was −0.58% (95% CI 0.01 to −1.17). Most of the secondary outcomes were significantly in favor of intensive treatment in both populations. However, both types of participants had a significant increase in serious adverse events, including hypoglycemic events.

The results of this review demonstrate that tight glycemic control is effective in preventing the development of DPN in patients with TIDM, but the data were not significant for patients with TIIDM (*p* = 0.06), although improved glucose control has been shown to significantly reduce nerve conduction and vibratory threshold abnormalities. The authors noted that this intervention significantly increases the risk of severe hypoglycemic episodes and should be taken into account when assessing risk/benefit. Buehler et al. [76], in 2013 published a systematic review and meta-analysis on the effect of tight glucose control compared to standard control, in this case in patients with TIIDM. It was determined that intensive glucose control significantly reduced the progression of retinopathy (RR 0.80; 95% CI 0.71–0.91), the incidence of DPN (RR 0.94; 95% CI 0.89–0.99), as well as the progression of nephropathy (RR 0.55; 95% CI 0.37–0.80) but had no significant effect on the incidence of nephropathy (RR 0.69; 95% CI 0.42–1.14). In agreement, Fullerton et al. [64] in 2014 conducted a systematic review in which it was observed that intensive glycemic control reduces the risk of developing microvascular complications compared to conventional treatment, in the case of neuropathy by 4.9% versus 13.9%; RR 0.35 (95% CI 0.23–0.53); *p* < 0.00001. Hasan et al. [77] in 2016 conducted a systematic review and meta-analysis evaluating the efficacy and safety of intensive control compared to standard glycemic control in preventing the development of diabetic foot. Intensive control with an HbA1c target of 6.0–7.5% was associated with a significant decrease in the relative risk of amputation (RR, 0.65; 95% CI, 0.45–0.94; I(2) = 0%). Intensive control was associated with a slower decrease in the sensitive vibration threshold (mean difference, L8, 27; 95% CI, L9, 75 to L6, 79). No effect on neuropathic changes (RR, 0.89; 95% CI, 0.75–1.05; I(2) = 32%) or ischemic changes (RR, 0.92; 95% CI, 0.67–1.26; I(2) = 0%) was found in nine RCTs of patients with TIIDM.

The management of glycemic control suggested an optimal therapeutic approach depending on the patients with TIDM and TIIDM. Despite adequate blood glucose control, patients with TIIDM are likely to develop neuropathy [72]. This is why, in patients with TIDM, glycemic control with an HbA1c target of less than 6% is advised to prevent DPN and in the case of patients with TIIDM, glycosylated hemoglobin could range from 7.0–7.9%.

### 4.2. Use of Drugs

Pop-Busui et al. [60], in 2013, conducted a study where it was observed that glycemic control therapy with insulin sensitizers (IS) with metformin and thiazolidinediones (TZD) significantly reduced the incidence of DPN compared to insulin-providing therapies (IP) such as sulfonylureas, meglitinide or insulin. This result could be due to the anti-inflammatory, oxidative stress, lipid profile and weight improvement effects of TZDs and metformin, which would be coupled with the reduction in glycemia. However, no other studies have been published comparing the efficacy of the different drugs used for the treatment of DM in terms of the prevention and development of DPN.

With respect to liraglutide, Brock et al. [53], did not find a significant effect in terms of DPN prevention, although a decrease in proinflammatory cytokines was observed.

### 4.3. Lifestyle Modification

The most important and largest study on the prevention of the development of TIIDM was the Diabetes Prevention Program (DPP) [78], where participants at high risk of developing DM were divided into two groups, and both were compared with placebo groups. One group was metformin, with an administration of 850 mg twice daily, and the other group was lifestyle modification through programs of at least 7% weight loss and 150 min of physical activity per week. The intervention reduced the incidence of DM by 58% (95% CI, 48 to 66%) in the lifestyle modification group and by 31% (95% CI 17 to 43%t) in the metformin group compared with placebo, highlighting the greater benefit of lifestyle modification.

Supporting these results, an RCT, “China Da Qing Diabetes Prevention” [79], divided participants into three subgroups: diet, exercise, and diet plus exercise. Participants in the combined intervention group obtained a 51% (hazard ratio (HR) 0.49; 95% CI 0.33–0.73) lower incidence of diabetes during the active period and 43% (0.57; 0.41–0.81) during the subsequent 20 years of follow-up.

The relationship of these interventions in terms of preventing vascular microcomplications in DM was detailed in the studies of Diabetes Prevention Program Research Group et al. [57] in 2015 and Gong et al. [58] in 2011. In both studies, negative results were obtained for the prevention of DPN development by not preventing the advancement of microvascular complications: However, in the study by Gong et al., it did decrease the incidence of severe retinopathy by 47%.

In contrast, in the case of the 2017 Look AHEAD Research Group et al. [56] study, it was determined that the intensive lifestyle intervention group demonstrated a significant decrease in DPN.

### 4.4. Practice of Physical Exercise

Balducci et al. [80] in 2006 examined the effects of long-term physical training on the development of DPN in patients with TIDM and TIIDM through an RCT. Significant differences were found in the improvement of nerve conduction in the peroneal and sural nerves for the group that performed physical activity, so the study suggests that long-term aerobic exercise could prevent or modify the onset of the natural history of DPN. This improvement in peroneal nerve conduction velocity and an improvement in neuropathic symptoms was observed in the longitudinal observational study by Azmi et al. [81].

Singleton et al. [82], in 2014, demonstrated increased intraepidermal nerve fiber density (IENFD) (1.5 ± 3.6 vs. −0.1 ± 3.2 fibers/mm, *p* = 0.03) of the leg in a cohort of 100 patients with DM and without neuropathy who received a weekly structured and supervised exercise program (*n* = 60) compared to patients who only received lifestyle counseling (*n* = 40), followed for 1 year.

Several RCTs have been published with positive results in terms of improved DPN with physical exercise, such as those conducted by Song et al. [28] in 2011, Mueller et al. [31] in 2013, Dixit et al. [33] in 2016, Ahmad et al. [39] in 2019, Stubbs et al. [38] in 2019, Dixit et al. [54] in 2014, Gholami et al. [61] in 2018 and Gholami et al. [59] in 2020.

However, several systematic reviews and meta-analyses have been published in favor of exercise as a preventive factor in DPN in patients with TIIDM, although it is unclear whether this effect is due to the associated decrease in HbA1c percentage, or whether other currently unidentified factors are involved.

In 2017, Villafaina et al. [83] published a systematic review determining that improved heart rate variability during exercise may be an important factor to consider as prevention in DN and associated mortality in patients with TIIDM. In the same vein, Bhati-Pooja et al. [84] in 2018 conducted a systematic review on physical exercise practice and autonomic cardiac function in patients with TIIDM ascertaining that this strategy significantly improves nerve conduction. Gu et al. [85] in 2019 observed a positive influence of aerobic exercise on nerve function. In the case of DM associated with obesity, patients with DM who have to undergo bariatric surgery show an improvement in neuropathic symptoms [86].

### 4.5. Limitations of the Study

The review presents several limitations. Firstly, many of the studies analyzed present heterogeneity in outcome measures, while others studies report small sample size and short duration of follow-up. The authors have found that there is little evidence, and many knowledge gaps persist in the use of preventive alternatives; this should be considered. Furthermore, the risk of detection in eight included studies. In addition, in terms of the neuropathy evaluation technique and according to the literature consulted, there is variability, which is why it should be considered as another limitation.

## 5. Conclusions

According to the present review, DPN cannot be cured, so preventive measures are essential, with glycemic control being the main strategy. The preventive interventions studied included intensive or standard glycemic control, the use of drugs for glycemic control, lifestyle modifications and the practice of physical exercise. In the case of patients with TIDM, a clear benefit of intensive glycemic control with an HbA1c < 6% in the prevention of microvascular complications. In patients with TIIDM, standard glycemic control with an HbA1c between 7.0 and 7.9% is recommended and lifestyle modifications based on the practice of physical exercise, dietary control and control of cardiovascular risk factors are emphasized. Intensive glycemic control with insulin-sensitizing drugs is recommended in patients with TIDM, as well as lifestyle modifications in patients with TIIDM. The practice of moderate aerobic physical exercise is emerging as an important preventive factor in the development of neuropathy. More consistent studies are needed and with unification in the evaluation techniques that allow for consolidating some aspects of the knowledge of DPN. Therefore, the main principles of treatment for peripheral neuropathy are glycemic control, foot care, and pain management.

## Figures and Tables

**Table 1:** Potential studies excluded.

Reason for Exclusion	Authors
RCTs that specifically address treatment rather than prevention of DPN	Farvid et al., 2011 [27] Song et al., 2011 [28] Rizzo et al., 2012 [29] Lavery et al., 2012 [30] Mueller et al., 2013 [31] Ulbrecht et al., 2014 [32] Dixit et al., 2016 [33] Ziegler et al., 2016 [34] Sharoni et al., 2018 [35] Venkataraman et al., 2019 [36] López-Moral et al., 2019 [37] Stubbs et al., 2019 [38] Ahmad et al., 2019 [39] Shu et al., 2019 [40] Sari et al., 2020 [41]
Cohort studies not from RCTs	Müller-Stich et al., 2013 [42] Hur et al., 2013 [43] Cho et al., 2014 [44] Ishibashi et al., 2018 [45] O’Brien et al., 2018 [46] Yang et al., 2020 [47] Cárdenas et al., 2019 [48]
Cohort studies that do not specifically address the prevention of DPN, but from RCTs	Aroda et al., 2016 [49]
	Gaede et al., 2016 [50]
	Abraham et al., 2018 [51]
	Braffett et al., 2020 [52]

**Table 2:** Intraclass correlation coefficient. Evaluation of agreement between continuous measurements.

	Intraclass Correlation ^a^	95% Confidence Interval		F Test with True Value 0			
		Lower Bound	Lower Bound	Value	df1	df2	Sig.
Single Measures	0.997 ^b^	0.995	0.995	687.400	10	10	0.000
Average Measures	0.999 ^c^	0.995	1.000	687.400	10	10	0.000

Two-way mixed effects model where people’s effects are random and measures’ effects are fixed. ^a^ Type C intraclass correlation coefficients using a consistency definition—the between measure variance is excluded from the denominator variance. ^b^ The estimator is the same, whether the interaction effect is present or not. ^c^ This estimate is computed assuming the interaction effect is ab-sent, because it is not estimable otherwise.

**Table 3:** Scores of the investigators on the quality scales of the included studies.

Authors	Scale	Review 1	Review 2
Ismail-Beigi et al., 2010	CASpe	10/11	10/11
Charles et al., 2011	CASpe	6/11	6/11
Gong et al., 2011	STROBE	16/22	16/22
Pop-Busui et al., 2013	STROBE	17/22	17/22
Dixit et al., 2014	CASpe	11/11	11/11
Martin et al., 2014	STROBE	16/22	16/22
Diabetes Prevention Program Research Group et al., 2015	STROBE	17/22	17/22
Look AHEAD Research Group et al., 2017	CASpe	9/11	9/11
Gholami et al., 2018	CASpe	9/11	9/11
Brock et al., 2019	CASpe	11/11	11/11
Gholami et al., 2020	CASpe	9/11	9/11

**Table 5:** Main characteristics of the studies included.

Authors	Design	Participants (*N*)	Groups	Diabetes Type	Average Age (Years)	Duration of the Study	Interventions	Measured Results
Brock et al. (2019)	RCT, double-blind, placebo-controlled	39	IG (Liraglutide) *N* = 19 CG (placebo) *N* = 20	TIDM	50.4	32 weeks	Liraglutide Placebo	Changes in nerve potentials, proinflammatory cytokines, autonomic function and peripheral neurophysiological tests. MNSI
Charles et al. (2011)	RCT with parallel groups	1161	Routine Care (RC) *N* = 459 Intensive multifactorial treatment (IT) *N* = 702	TIIDM	59.9	6 years	IT: Education, medication and promotion of healthy lifestyle. CR: Danish recommendations for diabetes care.	AAI Vibration detection threshold (tuning fork) Light touch (SW)
Diabetes Prevention Program Research Group et al. (2015)	Cohort study of a parallel-group placebo-controlled RCT	2776	Placebo *N* = 935 Metformin *N* = 926 Lifestyle *N* = 915	TIIDM	51	15 years	Metformin Placebo Lifestyle	Diagnosis of diabetes HbA1c Albuminuria (Nephropathy) Fundus evaluation (Retinopathy) SW light touch (Neuropathy)
Dixit et al. (2014)	RCT of parallel groups	87	CG *N* = 47 (10 lost) EG *N* = 40 (11 lost)	TIIDM	CG: 59.45 EG: 54.40	8 weeks	EG: Moderate aerobic exercise, foot care education, healthy diet CG: Standard medical care, education	Motor and sensory nerve conduction studies in peroneal and sural nerves MDNS
Gholami et al. (2018)	RCT of parallel groups	24	Exercise *N* = 12 Control *N* = 12	TIIDM	CG: 43 ± 6.4 EG: 42 ± 4.6	12 weeks	Exercise: Running, walking or treadmill 3 times/week for 20–45 min. Control: Maintain usual level of physical activity.	Weight, BMI, % fat HbA1c Nerve conduction velocity (NCV) and nerve action potential amplitude (APAN) peoneal, tibial and sural nerves
Gholami et al. (2020)	RCT of parallel groups	31	CG *N* = 15 EG *N* = 16	TIIDM	52.8 ± 9.6	12 weeks	EG: Cycling exercises CG: Maintaining the usual level of physical activity	HbA1c Fasting glucose Flow mediated dilation (FMD), changes in intima-media thickness and basal diameter in superficial femoral artery, MDNS
Gong et al. (2011)	Cohort study of parallel-group RCTs	577	CG = *N* = 136 (42 lost) EG = *N* = 441 (135 lost)	TIIDM	CG 66.7 ± 9.2 EG 64.7 ± 9.3	20 years	EG: diet, exercise or diet + exercise CG: Regular medical care	Plasma glucose HbA1c, oral glucose tolerance test, Examination ocular fundus Inspection extremity lower limb AAI Light touch (SW)
Ismail-Beigi et al. (2010)	RCT of parallel groups	10,251	Intensive therapy *N* = 5128 Standard therapy *N* = 5123	TIIDM	62.2 ± 6	3.5 years	Intensive therapy: HbA1c < 6.0% Standard therapy: HbA1c 7.0–7.9%	Albuminuria Creatinine Fundus examination MNSI Vibratory sensitivity (tuning fork), light touch (SW)
Look AHEAD Research Group et al. (2017)	RCT of parallel groups	5145	Intensive lifestyle intervention (ILI) *N* = 2570 Diabetes support and education (DSE) *N* = 2575	TIIDM	58.7	11 years	ILI: 7% weight loss, reduced caloric intake, and increased physical activity DSE: Diabetes education focused on diet and exercise	MNSI Light touch (SW)
Martin et al. (2014)	Cohort study of a parallel-group RCT	1345	Intensive insulin therapy (INT) *N* = 687 Conventional insulin therapy (CON) *N* = 688	TIDM	33.6 ± 7	14 years	INT: insulin treatment aimed at near-normal glycemia. CON: insulin treatment according to current standards	Vibratory sensitivity Light touch (SW) MNSI Nerve conduction studies HbA1c
Pop-Busui et al. (2013)	Cohort study of a parallel-group RCT	2159	Insulin-sensitizing treatments (IS) *N* = 1080 Insulin-providing treatments (IP) *N* = 1079	TIIDM	62 ± 9	4 years	Insulin-sensitizing treatments Insulin-providing treatments	HbA1c, Duration of DM, Albuminuria Retinopathy Alcohol and tobacco consumption Blood lipids, Blood pressure, MNSI Prevalence of DPN

## Data Availability

Details of the literature search are reported in Appendix A and Appendix B. Extracted data and details of the risk of bias assessments are available from the authors upon request.

## Appendix A. Search Strategy

### Appendix A.1. PubMed

#### Appendix A.1.1. Clinical Trials

Free search: (diabet* neuropath*) AND (“prevention” OR “control”) NOT (“ulcer” OR “wound” OR “cardiovascular” OR “pain” OR “nephropath*” OR “retinopath*” OR “protocol”)—Filters “Clinical trial”, “10 years” and “human” were applied: 74 results were obtained.

Search by descriptors: “Diabetic Neuropathies/prevention and control” [Mesh].

Clinical trial”, “10 years” and “human” filters were applied: 48 results were obtained.

Unified search: “Diabetic Neuropathies/prevention and control” [Mesh] OR [(diabet* neuropath*) AND (“prevention” OR “control”) NOT (“ulcer” OR “wound” OR “cardiovascular” OR “pain” OR “nephropath*” OR “retinopath*” OR “protocol”)].

The filters “Clinical trial”, “10 years” and “human” were applied: In this PubMed search, we obtained a total of 122 results, and with the unified search we obtained 84, eliminating 38 duplicate records.

#### Appendix A.1.2. Cohort Studies

Free search: (diabet* neuropath*) AND (“prevention” OR “control”) NOT (“ulcer” OR “wound” OR “cardiovascular” OR “pain” OR “netinopathy*” OR “retinopathy*” OR “systematic review” OR “meta-analysis”) AND (“cohort stud*” OR “cohort analysis”).

The filters “10 years” and “human” were applied: 40 results were obtained.

Search by descriptors: “Diabetic Neuropathies/prevention and control” [Mesh] AND (“cohort stud*” OR “cohort analysis”).

The filters “10 years” and “human” were applied: 20 results were obtained.

Unified search: (diabet* neuropath*) AND (“prevention” OR “control”) NOT (“ulcer” OR “wound” OR “cardiovascular” OR “pain” OR “netinopathy*” OR “retinopathy*” OR “systematic review” OR “meta-analysis”) OR [“Diabetic Neuropathies/prevention and control” [Mesh]] AND (“cohort stud*” OR “cohort analysis”).

The filters “10 years” and “human” are applied: in this search, we obtained a total of 60 results, and with the unified search 56 were obtained, so four duplicate records were eliminated.

### Appendix A.2. Scopus

Free search: (“diabet* neuropath*”) AND (“prevention” OR control).

This is limited to the last 5 years, from 2015 to 2020, with “article”, in the “keywords” section. We limited the search to “randomized controlled trial”, “Cohort Studies” and “human”.

The terms “diabetic nephropathy”, “case control studies”, “qualitfy of life”, “incidence”, “cerebrovascular accident”, “cardiovascular risk”, “cardiovascular disease”, “diabetic nephropathies”, “peripheral occlusive artery disease”, “child”, “autonomic neuropathy”, “case report”, “diagnostic imaging”, “coronary artery disease”, “depression”, “practical guideline”, “neuropathic pain”, “healthcare cost”, “pilot study” and “diabetic retinopathy” were excluded.

Search strategy: KEY((“diabet* neuropath*”) AND (“prevention” OR control)) AND DOCTYPE (ar) AND PUBYEAR > 2014 AND (LIMIT-TO (EXACTKEYWORD,”Human”) OR LIMIT-TO (EXACTKEYWORD,”Cohort Studies”) OR LIMIT-TO (EXACTKEYWORD,”Randomized Control Trial”)) AND (EXCLUDE (EXACTKEYWORD,”Case Control Study”) OR EXCLUDE (EXACTKEYWORD,”Diabetic Nephropathy”) OR EXCLUDE (EXACTKEYWORD,”Case-Control Studies”) OR EXCLUDE (EXACTKEYWORD,”Cardiovascular Disease”) OR EXCLUDE (EXACTKEYWORD,”Quality Of Life”) OR EXCLUDE (EXACTKEYWORD,”Incidence”) OR EXCLUDE (EXACTKEYWORD,”Cerebrovascular Accident”) OR EXCLUDE (EXACTKEYWORD,”Diabetic Nephropathies”) OR EXCLUDE (EXACTKEYWORD,”Peripheral Occlusive Artery Disease”) OR EXCLUDE (EXACTKEYWORD,”Child”) OR EXCLUDE (EXACTKEYWORD,”Autonomic Neuropathy”) OR EXCLUDE (EXACTKEYWORD,”Cardiovascular Risk”) OR EXCLUDE (EXACTKEYWORD,”Case Report”) OR EXCLUDE (EXACTKEYWORD,”Diagnostic Imaging”) OR EXCLUDE (EXACTKEYWORD,”Coronary Artery Disease”) OR EXCLUDE (EXACTKEYWORD,”Depression”) OR EXCLUDE (EXACTKEYWORD,”Practice Guideline”) OR EXCLUDE (EXACTKEYWORD,”Neuropathic Pain”) OR EXCLUDE (EXACTKEYWORD,”Health Care Cost”)).

In this database, the unified search could be performed directly, since filters were added. A total of 233 results were obtained.

### Appendix A.3. The Cochrane Library

Search strategy in “advanced search”: “diabetic neuropathy” AND (“prevention” OR “control”) NOT (“ulcer*” OR “wound*” OR “cardiovascular” OR “pain*” OR “nephropath*” OR “retinopath*” OR “treatment*” OR “protocol”).

The filters from 2010 to present and “trials” are added: 154 results were obtained.

Cohort studies could not be found in this search engine, since it only indexes RCTs and systematic reviews.

In this database, we manually selected the duplicates that appeared, since it collects records from other databases, and, consequently, there were eight duplicates.

### Appendix A.4. CINAHL

#### Appendix A.4.1. Clinical Trials

Search strategy: (“diabet* neuropath*”) AND (“prevention” OR “control”).

Filters applied: “Search all my search terms” “apply related words” “apply equivalent subjects”, limit publication date from 2010 to 2020, publication type “clinical trial”, “excludes pre-CINAHL”, and gender “all”: 26 results were obtained.

#### Appendix A.4.2. Cohort Studies

Strategy: (“diabet* neuropath*”) AND (“prevention” OR “control”)) AND (“cohort study” OR “cohort analysis”).

Filters applied: limit publication date from 2010 to 2020, “Search all my search terms” “apply related words” “apply equivalent subjects”, “exclude pre-CINAHL”, and gender “all”: 13 results were obtained.

Subsequently, all the references resulting from the search in all the databases were added to the bibliographic manager to eliminate possible duplicates between them, obtaining 13 more duplicates, which were eliminated. Finally, a total of 203 duplicates were eliminated.

## Appendix B. Individual Characteristics of the Studies Included in the Review

- Study 1: Brock et al., 2019 [53]

Methods: Double-blind, parallel-group, placebo-controlled RCT.

Participants: Adults with TIDM and confirmed symmetrical polyneuropathy. Thirty-nine participants were randomized to receive liraglutide (*N* = 19) or placebo (*N* = 20).

Interventions: To test whether long-term treatment with liraglutide (an injectable drug used for the treatment of diabetes and obesity, acting in the same way as incretins), induces a decrease in inflammation, thus improving neuronal function, and consequently diabetic neuropathy. The duration was 6 weeks with a dose of 1.2 mg/day, continuing until 26 weeks, for a total of 32 weeks.

Results: The primary endpoint was change in latency of early brain evoked potentials. Secondary endpoints were changes in proinflammatory cytokines, cortical evoked potentials, autonomic function and peripheral neurophysiological tests. Compared to placebo, liraglutide reduced interleukin-6 (*p* = 0.025) with similar reductions in other proinflammatory cytokines. However, neuronal function was not altered at the central, autonomic or peripheral levels. Treatment was associated with 3.38 kg (*p* < 0.001) of weight loss and a decrease in urine albumin/creatinine ratio (*p* = 0.02).

Conclusions: The study concluded that treatment with liraglutide reduced interleukin-6 in adults with TIDM but did not improve established DPN. Lowering the systemic level of proinflammatory cytokines could lead to the prevention or treatment of the neuroinflammatory component in the early stages of diabetic neuropathy.

- Study 2: Charles et al., 2011 [62]

Methods: Parallel-group RCT examining the effects of early detection and intensive multifactorial treatment (IT) of patients with TIIDM in primary care on the prevalence of DPN and PAD over 6 years.

Participants: The study sample of 1161 participants was divided into two groups, the routine care group, RC (*N* = 459) and the intensive multifactorial treatment group, IT (*N* = 702).

Interventions: The interventions employed were different for the groups, consisting in the IT group of physician and patient education, medication use and promotion of healthy lifestyle, control of hyperglycemia, blood pressure and cholesterol, according to the regimen used in the Steno-2 Study [70], and in the CR group, patients received the standard pattern of diabetes care according to the Danish national recommendations.

Results: No statistically significant effect of IT on the prevalence of DPN and PAD was found compared to CR. The prevalence of an AAI ≤ 0.9 was 9.1% (95% CI 6.0–12.2) in the CR group and 7.3% (5.0–9.6) in the IT group. In participants evaluated for vibration detection threshold and light touch sensation the prevalence of at least one abnormal test was 34.8% (26.7–43.0) in the CR group and 30.1% (24.1–36.1) in the IT group.

Conclusion: It was determined that in a population with patients with type 2 diabetes screen-detected, screening followed by IT was not found to lead to a statistically significant difference in the prevalence of DPN and PAD 6 years after diagnosis. Additional information: also called “ADDITION-Denmark”study.

- Study 3: Diabetes Prevention Program Research Group et al., 2015 [57]

Methods: Study of the 3-year Diabetes Prevention Program (DPP) [87] RCT surviving cohort.

Participants: All participants were offered lifestyle training at the end of DPP. Overall, 2776 (88%) of the surviving DPP cohort were followed in the DPP Outcomes Study (DPPOS 2002–2013) and were analyzed by intention-to-treat.

Interventions: The 1996–2001 DPPOS was an RCT comparing an intensive lifestyle intervention or masked metformin with placebo in a cohort selected to be at high risk of developing diabetes. During DPPOS, the lifestyle group received a semiannual booster and the metformin group received unmasked metformin. This research aimed to determine the long-term extent of the beneficial effects of the lifestyle intervention or metformin on diabetes prevention originally demonstrated in the DPP and whether diabetes-associated microvascular complications would be reduced.

Results: During 15 years of follow-up, lifestyle intervention and metformin reduced diabetes incidence rates by 27% (*p* < 0.0001) and 18% (*p* = 0.001), respectively, compared to the placebo group. At year 15, the cumulative incidence of DM was 55%, 56% and 62%, respectively. The end-of-study prevalence of the aggregate microvascular outcome, composed of nephropathy, neuropathy and retinopathy, was not significantly different between treatment groups (11–13%) compared to the overall cohort. However, in women (*n* = 1887), the lifestyle intervention was associated with a lower prevalence (8.7%) than in the placebo (11%) and metformin (11.2%) groups, with a 21% (*p* = 0.03) and 22% (*p* = 0.02) reduction in the lifestyle group compared to placebo and metformin, respectively. Compared to participants who progressed to DM, those who did not do so had a 28% lower prevalence of microvascular complications (*p* < 0.0001).

Conclusion: This study claims that lifestyle intervention or metformin significantly reduced the development of DM over 15 years in predisposed cohorts, although there were no overall differences in aggregate microvascular outcome between treatment groups. However, those who did not progress to DM had a lower prevalence of microvascular complications than those who did.

- Study 4: Dixit et al., 2014 [54]

Methods: Parallel group RCT. The authors proposed evaluating the effect of moderate-intensity aerobic exercise (40–60% of heart rate reserve) on DPN. Participants: Patients with TIIDM and clinical neuropathy, defined with a minimum score of 7 on the Michigan Diabetic Neuropathy Scale (MDNS). An experimental group (*N* = 47) and a control group (*N* = 40) were included.

Interventions: The experimental group (*N* = 47) received guidelines for moderate-intensity aerobic exercise, accompanied by standard medical care, foot care education and individual dietary recommendations. The control group (*N* = 40) received only standard medical care, foot care education and dietary recommendations.

Results: The groups suffered losses of 10 and 11 participants, respectively. Measurements were performed at baseline and at 8 weeks, including nerve conduction studies in the peroneal motor and sural sensory nerve, as well as the MDNS score. For the peroneal nerve, regarding nerve conduction velocity there was a significant difference in the two groups at 8 weeks (*p* = 0.03). This difference was also observed at 8 weeks in the sural sensory nerve, (*p* = 0.00). Significant differences were observed in the mean MDNS scores in the two groups at 8 weeks (*p* < 0.05).

Conclusion: It was established that moderate-intensity aerobic exercise may play a valuable role in interrupting the normal progression of MDNS in patients with TIIDM.

- Study 5: Gholami et al., 2018 [61]

Methods: Parallel group RCT. The study set out to examine the effects of aerobic training on nerve conduction velocity and action potential amplitude in lower limbs.

Participants: Men patients with TIIDM and DPN, 24 volunteers randomized into two groups: exercise group (*N* = 12) and control group (*N* = 12).

Interventions: Aerobic training consisted of 20–45 min walking or running at 50–70% of the heart rate reserve in three sessions per week for 12 weeks. Before and 48 h after the experimental period, nerve conduction studies were performed and blood samples were taken to analyze HbA1c, and fasting and 2 h postprandial glucose concentration.

Results: Sural nerve sensory conduction velocity (SNV) in the exercise group was significantly increased (from 35.2 ± 4.3 m/s to 37.3 ± 6.2 m/s) compared to the control group (*p* = 0.007). Changes in motor NCV in peroneal and tibial nerves and action potential amplitude (APAN) in all nerves studied were not significant between groups (*p* > 0.05). In addition, HbA1c decreased to a greater extent in the exercise group compared to the control (*p* = 0.014).

Conclusion: It was determined that aerobic exercise training may have the potential to hinder DPN progression by improving NCV. Given the scarce evidence in this domain, related to exercise, the mechanisms should be studied in the future.

- Study 6: Gholami et al., 2020 [59]

Methods: Parallel-group RCT. In relation to the previous study, in this case, the investigators evaluated the effect of physical training on superficial femoral artery (SFA) measurements and neuropathic symptoms in patients with DPN to observe the relationship of DPN with PAD.

Participants: Thirty-one volunteers with established DPN randomly assigned to the experimental (*N* = 16) and control (*N* = 15) groups.

Interventions: The experimental group performed cycling exercise (50–70% of heart rate reserve, 30–45 min, three sessions/week) for 12 weeks. Before and 48 h after the experimental period a 5-min flow-mediated dilation (FMD) response, changes in intima media thickness and basal diameter in SFA using color Doppler ultrasound and neuropathic score in MDNS were assessed as primary outcomes, and fasting glucose level, HbA1c and neuropathic score as secondary outcomes.

Results: The FMD percentage increased significantly in the experimental group (from 3.2 ± 1.1% to 5.7 ± 1.2%) compared to the control condition (*p* = 0.0001). However, there were no significant alterations in the basement membrane diameter and intima media thickness (*p* < 0.05). Significant improvements in fasting glucose, HbA1c and Michigan diabetic neuropathy score (MDNS) after exercise intervention (all *p* < 0.05) were also observed. Linear regression analysis indicated that the change in MDNS was significantly associated with change in HbA1c (*p* = 0.001) and FMD (*p* = 0.001).

Conclusion: This finding may be clinically of great importance, as metabolic and vascular factors have been indicated to be involved in the development of DPN.

- Study 7: Gong et al., 2011 [58]

Methods: Cohort study of participants in a parallel-group RCT. A 20-year follow-up study of the original participants was conducted to compare the incidence of microvascular complications in the combined intervention group versus the control group.

Participants: The original RCT involving 577 adults with impaired glucose tolerance (IGT) who were randomly assigned to a control group or the lifestyle intervention group (divided into three subgroups: diet, exercise, and diet plus exercise). Follow-up information was obtained on 542 (94%) of the original 577 participants.

Interventions: The aim of the diet intervention was to increase vegetable intake and reduce alcohol and sugar consumption of the participants and, in those who were overweight or obese, to reduce total calorie intake in order to lose weight. In the case of the exercise intervention, this consisted of increasing leisure time physical activity. The interventions were carried out over 6 years.

Results: The cumulative incidence of severe retinopathy was 9.2% in the combined intervention group and 16.2% in the control group (*p* = 0.03). After clinical and age adjusting, the incidence of severe retinopathy was 47% lower in the intervention group than in the control group (*p* = 0.048). No significant differences were found in the incidence of severe nephropathy (*p* = 0.96) or in the prevalence of neuropathy (*p* = 0.89) among survivors after 20 years.

Conclusion: Lifestyle intervention over 6 years in persons with IGT was associated with a 47% reduction in the incidence of severe vision-threatening retinopathy over a 20-year interval, mainly due to the lower incidence of diabetes in the intervention group. However, no similar benefits were observed for nephropathy or neuropathy. Additional information: also called “China Da Qing Diabetes Prevention Outcome Study”.

- Study 8: Ismail-Beigi et al., 2010 [55]

Methods: A parallel-group RCT, called the Action to Control Cardiovascular Risk in Diabetes (ACCORD) study [72], aimed to determine whether lowering blood glucose levels reduced the rate of microvascular complications in patients with TIIDM.

Participants: Patients with DM and with high HbA1c concentrations (>7.5%) and cardiovascular disease (or two or more cardiovascular risk factors) were randomized by central randomization to the intensive (target HbA1c of <6.0%) or standard (7.0–7.9%) glycemic control group. 10,251 patients were randomized, (*N* = 5128) to the intensive glycemic control group and (*N* = 5123) to the standard group.

Interventions: Intensive glycemic control compared with standard glycemic therapy. In this analysis, the predefined composite outcomes were: dialysis or renal transplantation, high serum creatinine (>291.7 μmol/L) or retinal photocoagulation or vitrectomy (first composite outcome), or peripheral neuropathy plus the first composite outcome (second composite outcome). Thirteen secondary measures of renal, ocular, and peripheral nerve function were also assessed. Investigators and participants were aware of the treatment group assignment. An analysis was performed for all patients who were evaluated for the microvascular outcome on the basis of the treatment assignment, regardless of treatments received or adherence to therapies.

Results: Intensive therapy was discontinued before the end of the study due to higher mortality in that group, and patients transitioned to standard therapy. At transition, the first composite outcome was recorded in 443 of 5107 patients in the intensive group versus 444 of 5108 in the standard group (HR 1.00, 95% CI 0.88–1.14; *p* = 1.00), and the second composite outcome was observed in 1591 of 5107 versus 1659 of 5108 (0.96, 0.89–1.02; *p* = 0.19). The results were similar at the end of the study, first composite outcome 556 of 5119 vs. 586 of 5115 (HR 0.95, 95% CI 0.85–1.07, *p* = 0.42); and the second 1956 of 5119 vs. 2046 of 5115, respectively (0.95, 95% CI 0.89–1.01, *p* = 0.12)). Intensive therapy did not reduce the risk of microvascular outcomes, but delayed the onset of albuminuria. Six secondary end-of-study measures favored intensive therapy (*p* < 0.05).

Conclusion: The research concludes that the microvascular benefits of intensive therapy must be weighed against increased total and cardiovascular disease-related mortality, weight gain, and high risk of severe hypoglycemia, so as ACCORD proved, intensive glycemic control therapy does not provide significant benefits in patients with TIIDM.

- Study 9: Look AHEAD Research Group et al., 2017 [56]

Methods: The study Look AHEAD (Action for Health in Diabetes) [88] was a parallel-group RCT. It examined whether the intensive lifestyle intervention weight loss decreased cardiovascular morbidity and mortality in overweight or obese adults with TIIDM. Due to the nature of the study, patients and center investigators were not blinded. In addition, the coordinating center staff members responsible for data management and statistical analysis were also not blinded.

Participants: Beginning in 2001, a total of 5145 overweight and obese individuals with TIIDM were randomized to intensive intervention (ILI) (*N* = 2570) or diabetes support and education (DSE) control group (*N* = 2575) using a web-based management system at the study coordinating center at Wake Forest School of Medicine (Winsto-Salem, NC, USA). Randomization was stratified by clinical center and was not disclosed to clinical staff responsible for obtaining data on study outcomes.

Interventions: Intensive intervention (ILI) or diabetes support and education (DSE) control group. Interventions ended in September 2012, 9–11 years after randomization, but both groups continued to be followed for primary and secondary outcomes. Neuropathy assessments included MNSI completed at baseline in all participants, 5145 (ILI *N* = 2570; DSE *N* = 2575) and repeated annually thereafter, as well as SW monofilament testing performed in 3775 participants (ILI *N* = 1905, DSE *N* = 1870) at 1 and 2.3 years after intervention discontinuation.

Results: At baseline, the MNSI questionnaire scores were 1.9 ± 0.04 and 1.8 ± 0.04 in the ILI and DSE groups, respectively (difference not statistically significant). After 1 year, when weight loss was maximal in the ILI group (8.6 ± 6.9%) compared with DSE (0.7 ± 4.8%), the respective MNSI scores were 1.7 ± 0.04 and 2.0 ± 0.04 (*p* ≤ 0.001). Subsequently, scores increased gradually in both groups, but remained significantly lower in the ILI group during the first 3 years and at the end of follow-up. In both groups, there was a significant association between the MNSI scores and changes in body weight, HbA1c and plasma lipids. There was no significant difference between groups in participants with MNSI physical examination scores ≥2.5, considered indicative of DN. Light tactile sensation measured separately in the right and left great toes did not differ between ILI and DSE, but when the data were combined for both toes, a light touch was better preserved in the ILI group.

Conclusion: It was determined that the ILI group had a significant decrease in DPN based on questionnaire diagnosis, which was associated with the magnitude of weight loss. In both the ILI and DSE groups, changes in MNSI score were also associated with changes in HbA1c and lipids. There were no significant effects of ILI on DPN physical examination measures performed 1–2.3 years after completion of the active intervention, except for light touch sensation, which was significantly better in the ILI group when measures were combined for both toes.

- Study 10: Martin et al., 2014 [15]

Methods: Surviving cohort study from the RCT Diabetes Control and Complications Trial and its follow-up study Epidemiology of Diabetes Interventions and Complications (DCCT/EDIC) [87,88]. The authors described the development and progression of neuropathy and related findings among patients with TIDM at 14 years after intervention.

Participants: Patients with TIDM. There were a total of 1441 (100%) DCCT participants, of whom 1375 (95.4%) agreed to participate in EDIC, of whom 1274 (92.7%) were active in year 13 in EDIC, of whom 1226 (96.2%) were evaluated for CAN and 1186 (93.1%) for DPN [89].

Interventions: Intensive glycemic control vs. standard control. The primary outcome of DPN was assessed by clinical symptoms, signs and results of nerve conduction studies during DCCT and repeated in EDIC in year 13/14. CAN was assessed by the R-R response to stimulated breathing, Valsalva ratio and blood pressure response during years 13/14 and 16/17. In addition, symptoms reflecting neuropathic pain and autonomic function (including hypoglycemia awareness) were collected annually in EDIC using standardized questionnaires; peripheral neuropathy was also assessed annually using the MDNS. Genitourinary function assessments were collected in EDIC year 10.

Results: Intensive therapy during DCCT significantly reduced the risk of DPN and CAN at the end of DCCT (64% and 45%, respectively, *p* < 0.01). The prevalence and incidence of DPN and CAN remained significantly lower in the intensive therapy DCCT group compared to the conventional therapy DCCT group until year 13/14 of EDIC [90].

Conclusion: It was established that the persistent effects of prior intensive therapy on neuropathy measures over 14 years of EDIC largely mirror those observed for other complications of DM. DCCT/EDIC provides important information on the influence of glycemic control and the clinical course of DN and, most importantly, on how to prevent neuropathy in patients with TIDM.

- Study 11: Pop-Busui et al., 2013 [60]

Methods: Cohort study from the parallel-group RCT Bypass Angioplasty Revascularization Investigation 2 Diabetes (BARI 2D) [91] published in 2009. This trial demonstrated similar long-term clinical effectiveness of insulin-sensitizing (IS) versus insulin-providing (IP) treatments for patients with TIIDM on cardiovascular outcomes in a cohort with documented coronary artery disease.

Participants: A total of 2159 participants with TIIDM with documented coronary artery disease. IS (*N* = 1080), IP (*N* = 1079).

Interventions: Randomized glycemic control strategy of insulin-sensitizing (IS) versus insulin-providing (IP) treatments for TIIDM on the prevalence and incidence of DPN. DPN (defined as Michigan Neuropathy Screening Questionnaire (MNSI) > 2 clinical examination score) was assessed at baseline and annually for 4 years. Prevalence and incidence of DPN were compared by intention-to-treat models using generalized estimating equations logistic models for prevalence and Kaplan–Meier estimates and Cox regression models for incidence rates.

Results: The results were obtained for 2159 participants in the BARI 2D study (70% male) with baseline values and at least one follow-up MNSI score (mean age 62 ± 9 years, mean HbA1c 7.7 ± 1.6%, duration of diabetes 10 ± 9 years). There was no difference in the prevalence of DPN between the IS and IP groups during the 4 years of follow-up. In 1075 BARI 2D study participants without DPN at baseline, the 4-year cumulative incidence rate of DPN was significantly lower in the IS (66%) than in the IP strategy group (72%) (*p* = 0.02), which remained significant after adjusting for HbA1c (*p* = 0.04). In subgroup analyses, the IS strategy had a greater benefit in men (Hazard Ratio 0.75 [99% 95% CI 0.58–0.99], *p* < 0.01).

Conclusion: Among patients with TIIDM followed for up to 4 years in BARI 2D, a glycemic control therapy with IS significantly reduced the incidence of DPN compared to IP therapy and may provide more benefits for men.
